# Impact of Ebola virus nucleoprotein on VP40 virus-like particle production: a computational approach

**DOI:** 10.1038/s42003-024-06300-8

**Published:** 2024-05-25

**Authors:** Xiao Liu, Robert V. Stahelin, Elsje Pienaar

**Affiliations:** 1 Weldon School of Biomedical Engineering, Purdue University, West Lafayette, IN USA; 2 Department of Medicinal Chemistry and Molecular Pharmacology, Purdue University, West Lafayette, IN USA; 3 Regenstrief Center for Healthcare Engineering, Purdue University, West Lafayette, IN USA

**Keywords:** Dynamical systems, Ebola virus, Computational models, Viral infection

## Abstract

Ebola virus (EBOV) matrix protein VP40 can assemble and bud as virus-like particles (VLPs) when expressed alone in mammalian cells. Nucleoprotein (NP) could be recruited to VLPs as inclusion body (IB) when co-expressed, and increase VLP production. However, the mechanism behind it remains unclear. Here, we use a computational approach to study NP-VP40 interactions. Our simulations indicate that NP may enhance VLP production through stabilizing VP40 filaments and accelerating the VLP budding step. Further, both the relative timing and amount of NP expression compared to VP40 are important for the effective production of IB-containing VLPs. We predict that relative NP/VP40 expression ratio and time are important for efficient production of IB-containing VLPs. We conclude that disrupting the expression timing and amount of NP and VP40 could provide new avenues to treat EBOV infection. This work provides quantitative insights into EBOV proteins interactions and how virion generation and drug efficacy could be influenced.

## Abstract (web summary)

A computational modeling study indicates that relative amount and timing of Ebola virus proteins VP40 and NP drive effective viral particle production.

## Introduction

Ebola virus (EBOV) is one of the most fatal known pathogens since its discovery in 1976^1,2^. Over the last 40 years, more than 34,000 people have been infected and greater than 15,000 people have been killed in 44 known outbreaks^3,4^. While two antibody-based therapies were approved in 2021^5,6^, the mortality rate is still greater than 30% even with therapy. There’s a need to continue developing new treatment for EBOV and better understand potential strategies for small molecule treatments.

Our understanding of the subcellular dynamics of EBOV is still limited. Research with live EBOV must be conducted in biosafety level 4 (BSL-4) labs, which slows research progress. Further, EBOV contains seven multi-functional viral proteins, all with complex protein-protein interactions, making it difficult to identify effective drug targets. However, EBOV matrix protein VP40 can assemble and bud in the form of virus-like particles (VLPs) from the plasma membrane of mammalian cells^7,8^. This feature of VP40 makes it a useful system to study the assembly and budding process of EBOV in BSL-2 conditions.

Aside from the viral matrix, the nucleocapsid (NC), which encapsulates viral RNA, is another important structure in EBOV assembly^9^. The NC consists of at least NP, minor matrix protein (VP24), and polymerase cofactor (VP35)^10,11^. Apart from being wrapped by VP40, these NC-related proteins can modify the morphology and production efficiency of VP40 VLPs when co-expressed with VP40^12,13^. To fully understand the assembly and budding process of EBOV, the complex interactions between matrix proteins and NC proteins must be elucidated.

NP, the critical component of the NC, has complex interactions with VP24^10,14^, VP35^10,14^, VP30^15^, VP40^16,17^ and itself^11,18,19^. It is also an important component of the ribonucleoprotein (RNP) complex, which is responsible for transcription, replication, and protection of EBOV RNA^20,21^. When expressed by itself in mammalian cells, NP assembles into 20–25 nm diameter helical tubes, which have the same dimensions as the core structure of NCs^9,11,18^. These findings suggest that studying NP assembly, in the context of VP40 assembly, can provide vital insights into the assembly of the NC and its interaction with the viral matrix.

Previous work has shown that co-expression of NP enhances VP40 VLP production^13^. Though they found that the C-terminal domain (CTD) of NP is important for both increased VLP production as well as the recruitment of NP to VLPs, the detailed mechanisms of this influence of NP on VP40 VLP production and dynamics remains unclear. Other studies found that cytoplasmic NCs contains no detectable VP40s when moving to the plasma membrane^22^, and the rate of movement of NC in the cytoplasm is not affected by VP40 co-expression^10^. VP40 has the ability to recruit NCs to the site of budding on the cell membrane^10,22^. Together, these findings indicate that the interaction between VP40 and NP happens on the host cell plasma membrane. However, recent work suggested that the interaction between VP40 and NP takes place both in the cytoplasm and on the plasma membrane (two-stage interactions)^17^. These authors concluded that NP interacts with VP40 in the cytoplasm through NP’s N-terminal domain (NTD) and induces a conformational change in NP’s CTD which is responsible for the recruitment of NP to the cell membrane and the incorporation of NP IBs into VLPs. When NP CTDs were mutated and lost the ability to interact with VP40s, they observed that VP40s will be trapped in IBs in the cytoplasm, as plasma membrane localization and VLP production will be reduced. These unknows and seemingly conflicting results demonstrate the need for quantitative insights into NP-VP40 (or NP-VLP) interactions.

Mathematical modeling is a valuable tool to provide such quantitative insights into complex biological systems^23^. We previously developed the first ODE-based model of VP40 assembly and budding at the intracellular level^24,25^. Our model suggested several mechanisms of VP40 and phosphatidylserine (PS) interactions regarding the formation of VLPs, such as the influence of PS on VLP egress. We also revealed the dynamics of VP40 oligomers in the process of VLP assembly.

Here, we build on this prior work with VP40^24,25^ to construct an ODE-based model of EBOV NP-VP40 interactions, assembly and budding at the subcellular level. We use this model to (a) test which interactions between NP and VP40 can give rise to the experimentally observed impacts of co-expression, as well as (b) quantify the impact of NP on VP40 VLP production.

## Results

### An ODE-based model replicates the impact of NP on VP40 VLP budding through a two-stage interaction

**Fig. 1:**
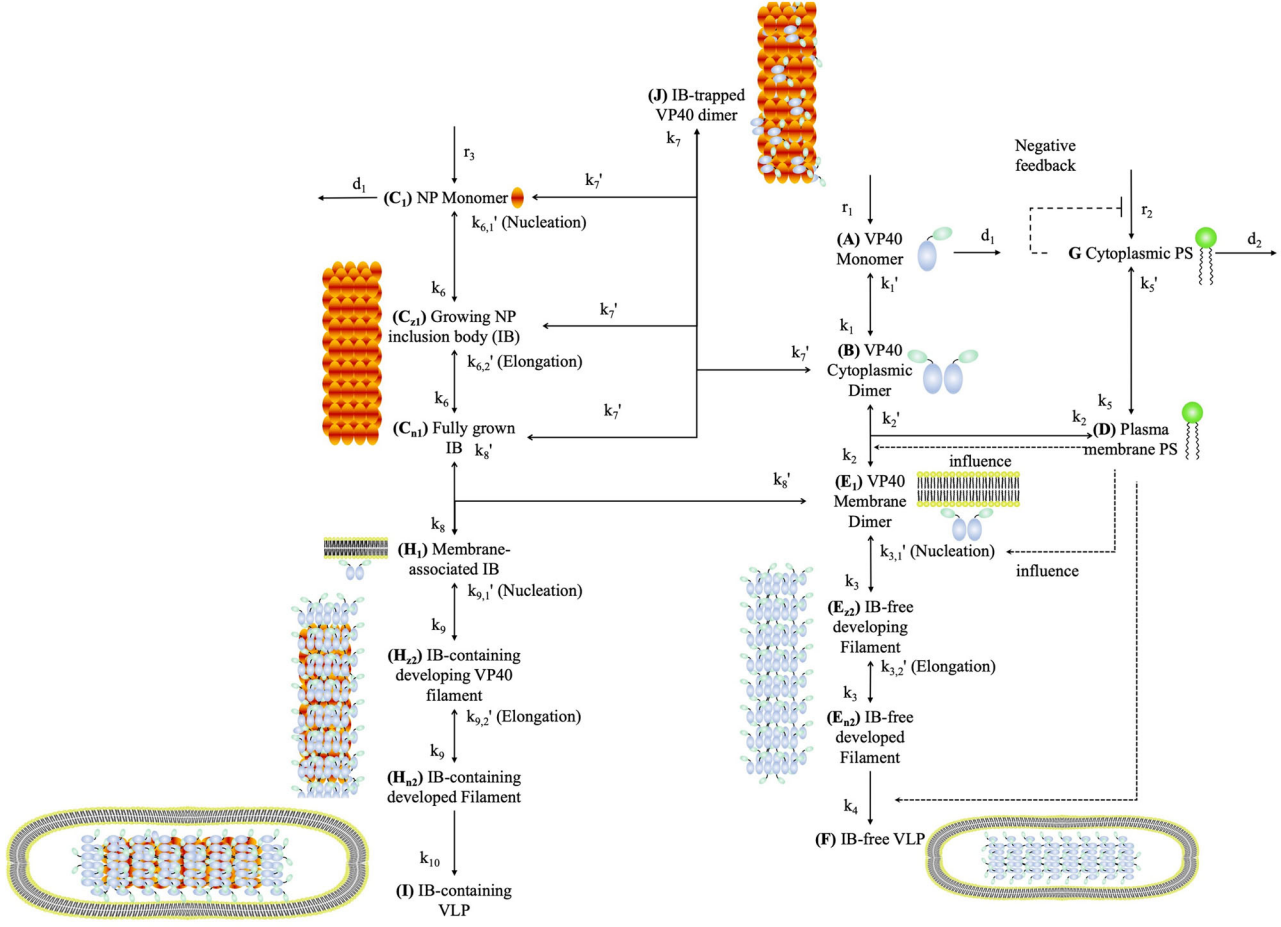
Scheme of the NP-VP40 model. The right side outlines the VP40 and PS model structure from prior work^24,25^; the left side outlines new model components relating to NP interactions. The VP40 model (right side) includes: VP40 monomer production (*r*_1_); VP40 monomer degradation (*d*_1_); reversible dimerization of VP40 monomers (forward rate *k*_1_, reverse rate *k*_1_’); reversible VP40 dimer association with host cell membrane PS (forward rate *k*_2_, reverse rate *k*_2_’); reversible oligomerization of VP40 dimers in a nucleation process (forward rate *k*_3_, reverse rate *k*_3,1_’); reversible oligomerization of VP40 dimers into mature filaments in an elongation process (forward rate *k*_3_, reverse rate *k*_3,2_’); and budding of mature empty VLPs from the host cell membrane (*k*_4_). This VP40 model is influenced by host cell PS levels (top right): cytoplasmic PS production (*r*_2_) and degradation (*d*_2_); cytoplasmic PS incorporation into the host cell membrane (forward rate *k*_5_, reverse rate *k*_5_’); and reversible association of membrane PS with cytoplasmic VP40 (forward rate *k*_2_, reverse rate *k*_2_’). Cytoplasmic PS has negative feedback on its own production (*r*_2_); and positive feedback on VP40 dimer membrane association (*k*_2_), reverse reaction of VP40 dimer oligomerization during nucleation (*k*_3,1_’), and VLP budding (*k*_4_). The NP-VP40 model (left side) includes NP monomer production (*r*_3_) and degradation (*d*_1_), reversible oligomerization of NP monomers in a nucleation process (forward rate *k*_6_, reverse rate *k*_6,1_’); reversible oligomerization of NP monomers into mature IBs in an elongation process (forward rate *k*_6_, reverse rate *k*_6,2_’); mature IBs binding to membrane associated VP40 dimers to become membrane associated IBs (forward rate *k*_8_, reverse rate *k*_8_’); membrane associated IBs producing IB-containing VP40 filaments (forward rate *k*_9_, reverse rate *k*_9_’); and budding of IB-containing VLPs (*k*_10_). In addition, cytoplasmic NP monomers and IBs bind and trap cytoplasmic VP40 dimers (forward rate *k*_7_, reverse rate *k*_7_’). Further details on model construction and equations can be found in the Materials and Methods section. Figure was generated in Microsoft Powerpoint using elements from our prior work^24,25^.

We developed our NP-VP40 model (Eqs. (1)–(27)) based on our previous model that was composed of VP40 interactions alone^24,25^. Here, we explicitly incorporated the assembly of NP IBs and the assembly and budding of VP40 filaments (with or without NP IBs). We also include the experimentally identified two-stage interaction between NP and VP40 by having cytoplasmic NP interacting with cytoplasmic VP40 dimer as well as full IBs interacting with membrane-associated VP40 dimers^10^ (Fig. 1). Details of the model structure are outlined in the figure caption. Our first aim was to determine if our model can replicate experimental data from literature using this two-stage interaction assumption^13,17^.

**Fig. 2:**
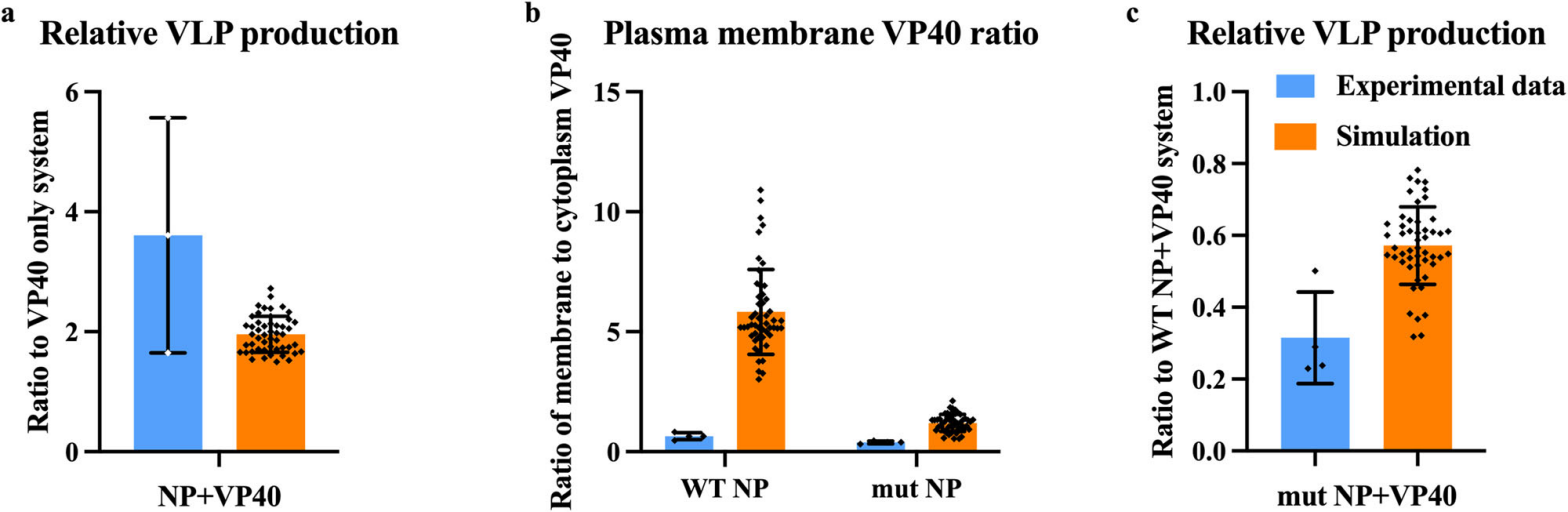
Simulation from NP-VP40 model reproduces experimental observations. Experimental data represented in blue bars were data extracted from the published figures using Adobe Photoshop and Microsoft Excel^13,17^, and is summarized in Methods. Orange bars represent our model simulation results that were calibrated to the experimental data shown. **a** Simulated relative VLP production at 24 h is increased 1.96-fold on average when NP is co-expressed comparing to VP40-only, which falls within experimental the range of 3.6 ± 1.96 at 24 to 30 h^13^. One sample *t* test results show that the simulations are significantly different from a ratio of 1 (*p* < 0.0001, *n* = 50). While the experimental ratio was not statistically significantly different from 1 (*p* = 0.15, *n* = 3), the sample size is small and all experimental data points are above 1. **b** Simulated plasma membrane VP40 ratio is 5.83 at 24 h when WT NP is co-expressed and significantly reduces to 1.2 when mutant NP is co-expressed (*p* < 0.0001, *n* = 50). This corresponds to ratios of 6.33 ± 1.55 and 0.64 ± 0.14 at 24 h in the experimental data (*p* = 0.01, *n* = 4), respectively^17^. **c** Simulated relative VLP production when mutant NP is co-expressed with VP40 is 0.57 of the value for WT NP at 36 h (*n* = 50, significantly different from 1, *p* < 0.0001). This aligns with the experimental observations that the mutant NP leads to VLP production that is 0.31 ± 0.13 of WT production at 36 h^17^ (*p* = 0.002, *n* = 4). Error bars indicate SD.

We identified a set of 50 parameter sets through model calibration to relevant experimental data. During calibration we allowed NP-related parameters to vary, while VP40-only parameters are randomly sampled from previously determined values^25^ (See Materials and Methods and Supplementary Data 1–2 for calibration details. Parameters that were sampled from previous values are marked in Supplementary Information Table S1). These calibrated parameter sets successfully reproduced key experimental data (Supplementary Data 3). Specifically, the addition of NP in our simulations reflected the experimentally observed increase in VLP production compared to VP40-only (Fig. 2a)^13^. While the membrane association-deficient NP mutant decreases both the membrane VP40 ratio and VLP production compared to WT NP (Fig. 2b, c), in agreement with experimental observations^17^. Experimental work also indicated that the interaction between VP40 and NP in the cytoplasm should be increased when NP is mutated^17^. In our model predictions, the increase (as indicated by P_1_, the portion of all cytoplasmic NP that is interacting with VP40 in the cytoplasm) is consistent but very small due to the high P_1_ in WT NP group (Supplementary Information Fig. S1, Supplementary Data 4). Thus, our simulations can reproduce the experimentally identified impacts of NP co-expression on VP40 VLP production.

As qualitative validation, we further tested our model predictions with the 50 parameter sets against the experimentally observed bimodal distribution of IB size when NP is expressed alone^26^. The work showed that the average size of IB will increase from 10 to 24 h, and both very small and large sized IBs are dominant, especially at later time points in a NP-only system^26^. In the experimental study, NP was detected after 10 h. However, it already becomes detectable in our model after 1 h. Since our model does not explicitly include a preparation stage for protein production, there is a time shift between our simulated system and the experimental observation, and the starting time of our simulation corresponds to approximately 9 h in their experiment. As a result, we evaluated IB size changes between 1 and 15 h in our model, instead of 10 to 24 h as in the experiments. Our prediction agrees with the experimental observation that while the average size of IB increases over time (Supplementary Information Fig. S2a, Supplementary Data 5), the distribution becomes bimodal after several hours as the system reaches steady state (Supplementary Information Fig. S2b, Supplementary Data 6). Based on these quantitative and qualitative calibration and testing between our model results and experimental data, we believe that our model can replicate important features of the NP-VP40 system.

**Fig. 3:**
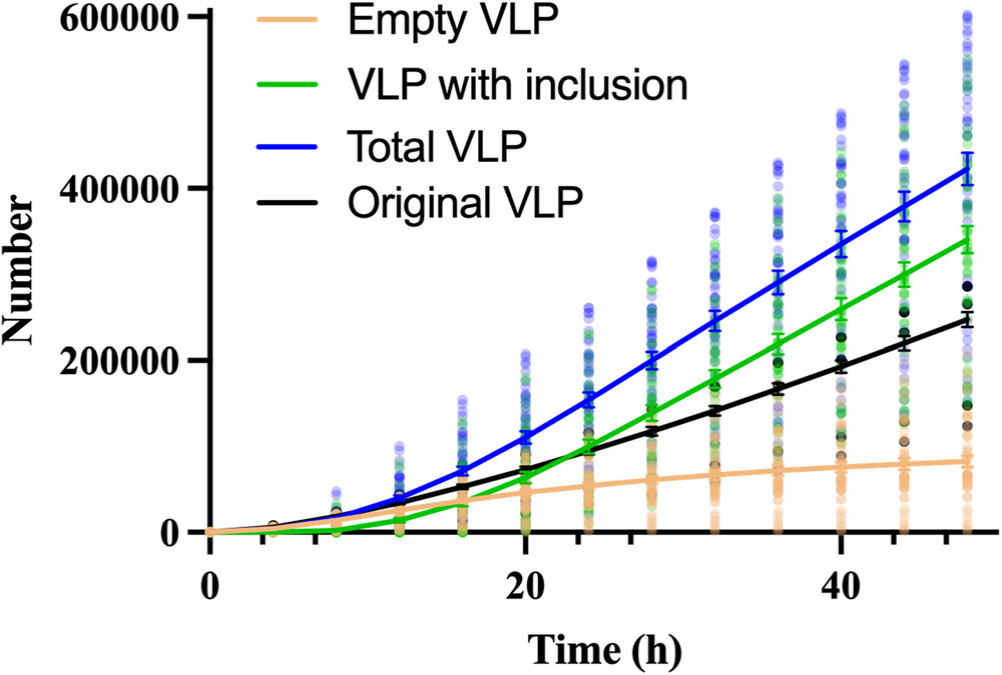
Simulation-predicted VLP production from NP-VP40 system. Total VLP production is increased during NP-VP40 co-expression compared to VP40-only, and the dominant form of VLPs is IB-containing VLP. On the other hand, IB-free VLP production is reduced compared to VP40-only system. Error bars indicate SEM.

We next used this calibrated and tested model to assess the impact of NP on VLP production in the 50 calibrated parameter sets. Our simulations indicate that when NP is co-expressed with VP40, the increase in total VLP production compared to the VP40-only system^25^ is attributed to a large amount of IB-containing VLPs. In fact, the IB-containing VLPs in the co-expression system outnumbers the total VLPs in the VP40-only system, while the IB-free VLP number is lower (Fig. 3, Supplementary Data 7). This suggests that co-expression of NP not only increases VLP production, but also prevents the formation of IB-free VLPs.

To better understand how NP is achieving this impact on VLP production, we next evaluate parameter differences in the VP40 and NP-VP40 model mechanisms.

### VP40 filament stability and VLP budding rate is positively impacted by NP

**Fig. 4:**
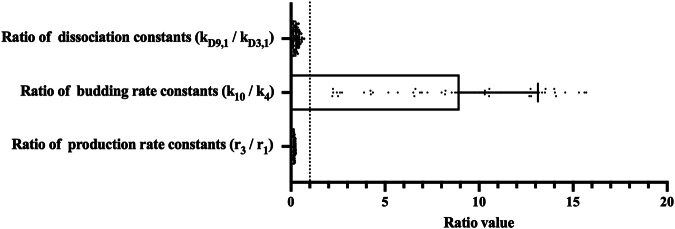
Range of important parameter ratios between NP + VP40 and VP40-only system. Our calibrated parameter values indicate that co-expression of NP decreases the dissociation constant for filament growth and increases VLP budding rate. Monomer production rate for NP is predicted to be much lower than VP40 in our system. Error bars indicate SD.

Since we calibrated both the VP40 and the NP-VP40 mechanisms, the resulting parameter estimates from both systems can give us insights into which VLP assembly mechanisms are predicted to be strongly impacted by NP. Our results identified three parameters that are different between the VP40 and NP-VP40 mechanisms (Fig. 4, Supplementary Data 8). First, we find that the dissociation constants for IB-containing filament growth (*KD*_9,1_) is lower than the dissociation constants for IB-free filament growth (*KD*_3,1_). This indicates that, since VP40 can interact with IBs, the stability of IB-bound filament is predicted to be higher than IB-free filaments. Second, the VLP budding rate for IB-containing VLPs (*k*_10_) is increased compared to the budding rate in IB-free VLPs (*k*_4_). This suggests that NP IB stabilization of the growing VP40 filaments also supports the budding process. Third, our model predicts that the production rate of NP (*r*_*3*_) is lower than the production rate for VP40 (*r*_1_). This indicates that NP expression levels need to be lower than VP40 in order to reproduce the experimental data.

Taken together, these parameter differences highlight how NP could affect VP40 filament growth and budding; and identify potential fundamental differences in NP and VP40 expression that supports VLP production. To further characterize the influence of each of these three parameters on both IB-containing and IB-free VLPs, we next perform local sensitivity analyses.

### NP differentially affects VLP production via its influence on filament stability and budding

**Fig. 5:**
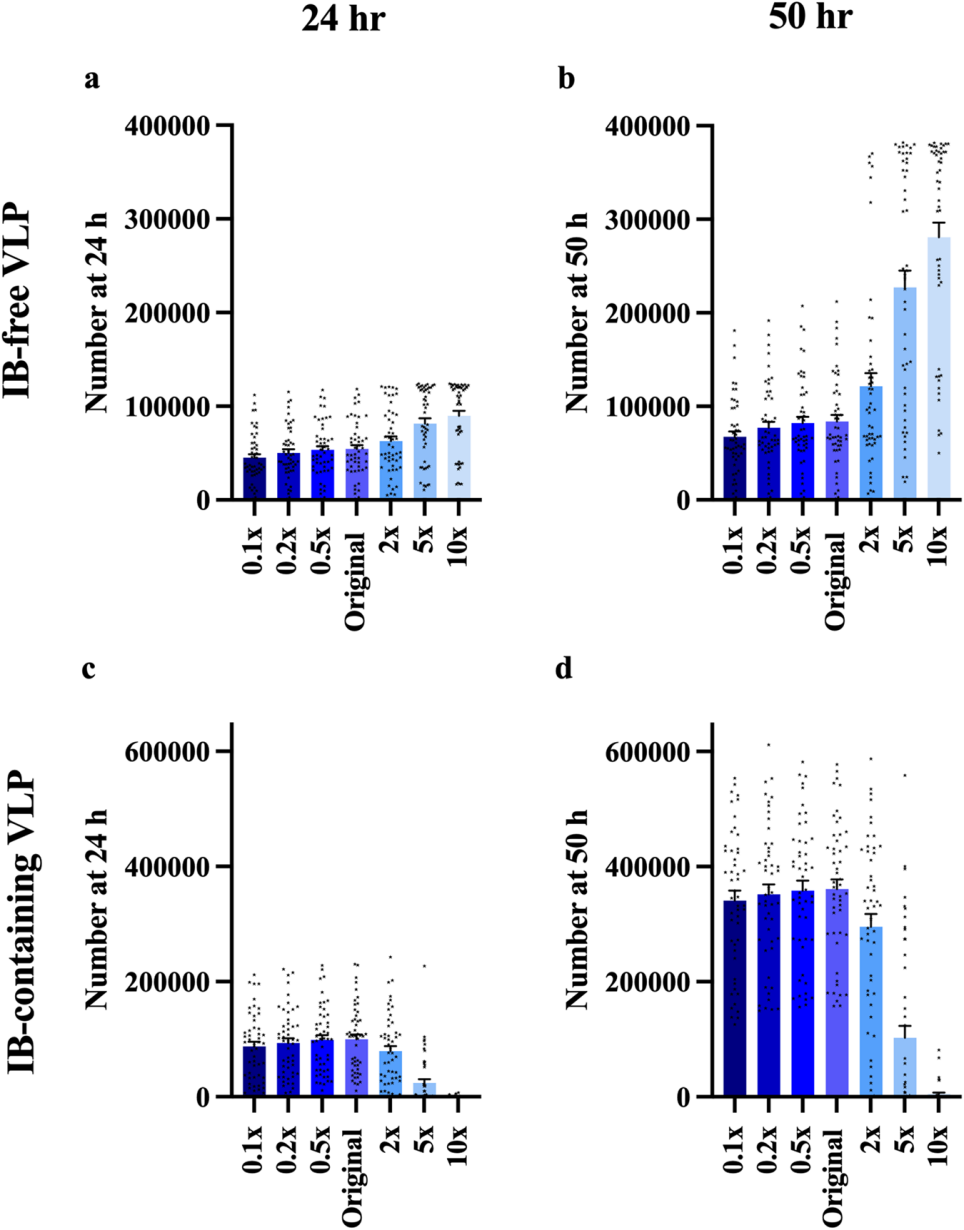
Simulation-predicted influence of IB-containing filament growth dissociation constant on VLP production. **a**, **b** IB-free VLP production elevates as k_D9,1_ and k_D9,2_ increase from 0.1× to 10×. **c**, **d** IB-containing VLP production decreases when k_D9,1_ and k_D9,2_ increases. The reduction under large k_D9,1_ and k_D9,2_ is more obvious. Error bars indicate SEM.

To characterize how the influence of NP on VLP filament stability and budding affects VLP production, we vary the parameters of interest (dissociation constants for IB-containing filament growth *KD*_9,1_, *KD*_9,2,_ and VLP budding rate constant *k*_10_) for each of our 50 parameter sets. As *KD*_9,1_ and *KD*_9,2_ increases, the number of IB-free VLPs also increases (Fig. 5a, b, Supplementary Data 7, 9) while the number of IB-containing VLPs decreases (Fig. 5c, d, Supplementary Data 7, 9). This can be expected as a higher dissociation constant for IB-containing filaments will make more VP40 dimers available for assembly into IB-free filaments.

**Fig. 6:**
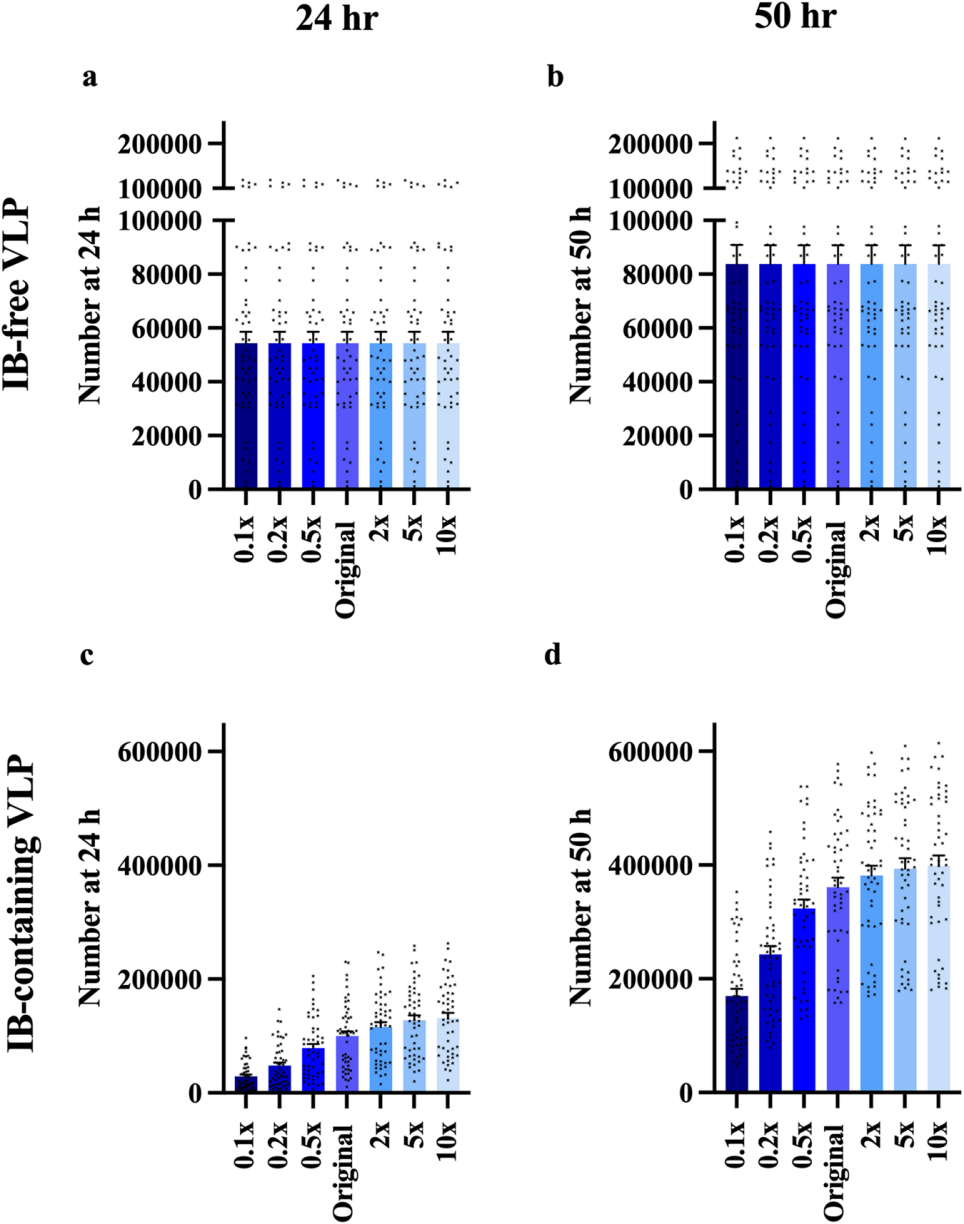
Simulation-predicted influence of IB-containing VLP budding rate on VLP production. **a**, **b** IB-free VLP production is not influenced by k_10_. **c**, **d** IB-free VLP production increases as k_10_ increases from 0.1× to 10×. Error bars indicate SEM.

To evaluate how NP impacts the VLP budding process we varied the value of the IB-containing VLP budding rate constant (*k*_10_). Unlike *KD*_9,1_ and *KD*_9,2_, the impact of *k*_10_ on IB-free VLP production is very limited (Fig. 6a, b, Supplementary Data 7, 10). On the other hand, IB-containing VLP production increases with *k*_10_ (Fig. 6c, d, Supplementary Data 7, 10). This indicates that changes in the budding rate of IB-containing VLPs does not affect the pool of VP40 available for assembly of IB-free VLPs, but it does affect how many IB-containing VLPs are produced.

Taken together, these results demonstrate that NP lowers the production of IB-free VLPs via its ability to stabilize growing IB-containing filaments (lower *KD*_9,1_ and *KD*_9,2_ compared to *KD*_3,1_ and *KD*_3,2_). In contrast, the NP-associated increase in production of IB-containing VLPs is associated with the ability of NP to both stabilize growing IB-containing filaments as well as promote VLP budding (higher *k*_10_ compared to *k*_4_).

### NP/VP40 expression ratio influences the production of IB-containing VLP production

**Fig. 7:**
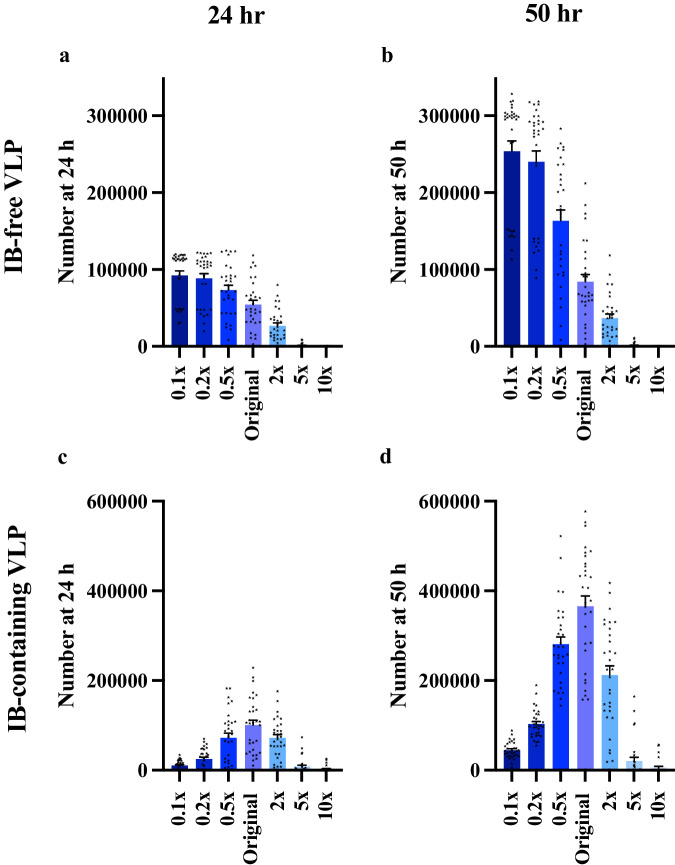
Simulation-predicted influence of NP production rate on VLP production. **a**, **b** IB-free VLP production decreases as r_3_ increases from 0.1× to 10×. **c**, **d** IB-containing VLP production decreases when r_3_ is either very small or large. Error bars indicate SEM. 31 out of 50 groups are used for analysis as others have met tolerance problems in ode-solver.

Our calibrated model indicated that NP production rates (*r*_3_) are lower than VP40 production rates (*r*_1_) (Fig. 4). To characterize how the NP/VP40 production ratio impacts VLP production, we vary the NP monomer production rate constant (*r*_3_). As the NP production rate (*r*_3_) increases, the production of IB-free VLP decreases, which is expected since NP can inhibit IB-free VLP production (Fig. 7a, b, Supplementary Data 7, 11). However, IB-containing VLP production is inhibited when NP production is both too high and too low (Fig. 7c, d, Supplementary Data 7, 11).

It is expected that that too little NP will provide insufficient IBs for IB-containing VLPs. To explain the inhibition of IB-containing VLPs at high NP production rates, we hypothesized that too much NP may trap large amounts of VP40 in the cytoplasm and prevent the formation of filaments, due to the two-stage interaction between NP and VP40. To test our hypothesis, we evaluated the amount of VP40 bound to cytoplasmic IB. We do find that more VP40s will be trapped in cytoplasmic IBs as NP production rate increases (Supplementary Information Fig. S3a, Supplementary Data 12). However, when we evaluate cell membrane VP40 number, though it is negatively related to NP production rate at the beginning, both low and high NP production rate will have more VP40 on the cell membrane (Supplementary Information Fig. S3b, Supplementary Data 12). This can be expected, since total VLP budded in those groups are low (Supplementary Information Fig. S3c, Supplementary Data 12), especially in high NP production groups (5×, 10× NP production rate), leaving more VP40s remaining on cell membrane. This suggests that, for high NP production rates, indeed some VP40 is getting trapped in the cytoplasm, but that this does not necessarily limit the amount of membrane-associated VP40, and therefore cannot explain why high NP production rates result in lower IB-containing VLP production.

Thus, the question remains why IB-containing VLPs are not being produced when membrane-associated VP40 concentration is sufficient. To answer this question, we further evaluated the concentration of filament building blocks (i.e., VP40 dimers). The cell membrane VP40 dimer concentration is very low in high NP production rate simulations (Supplementary Information Fig. S3d, Supplementary Data 12) while there are more IBs moving to the cell membrane in those groups (Supplementary Information Fig. S3e, Supplementary Data 12). These IBs moving to the cell membrane are less likely to “release” VP40 dimer since IB-containing filaments are more stable than IB-free filaments (Fig. 4, Supplementary Data 8). Thus, while the demand of cell membrane VP40 dimer is high for the maturation of those IB-containing filaments, the concentration of VP40 dimers is low, which leads to low IB-containing VLP production. Taken together, when NP production is too high, VP40 will be trapped in both cytoplasmic IB and incomplete IB-containing filaments that are unable to bud because of a lack of VP40 dimers needed to complete assembly and budding. Note that in this model, we assume that all NPs in the cytoplasm have the ability to interact with VP40. If this is not true, the optimum NP/VP40 expression rate for higher IB-containing VLP production can be much higher, but there should still be an optimum ratio.

### NP/VP40 expression timing influences the production of IB-containing VLP production

**Fig. 8:**
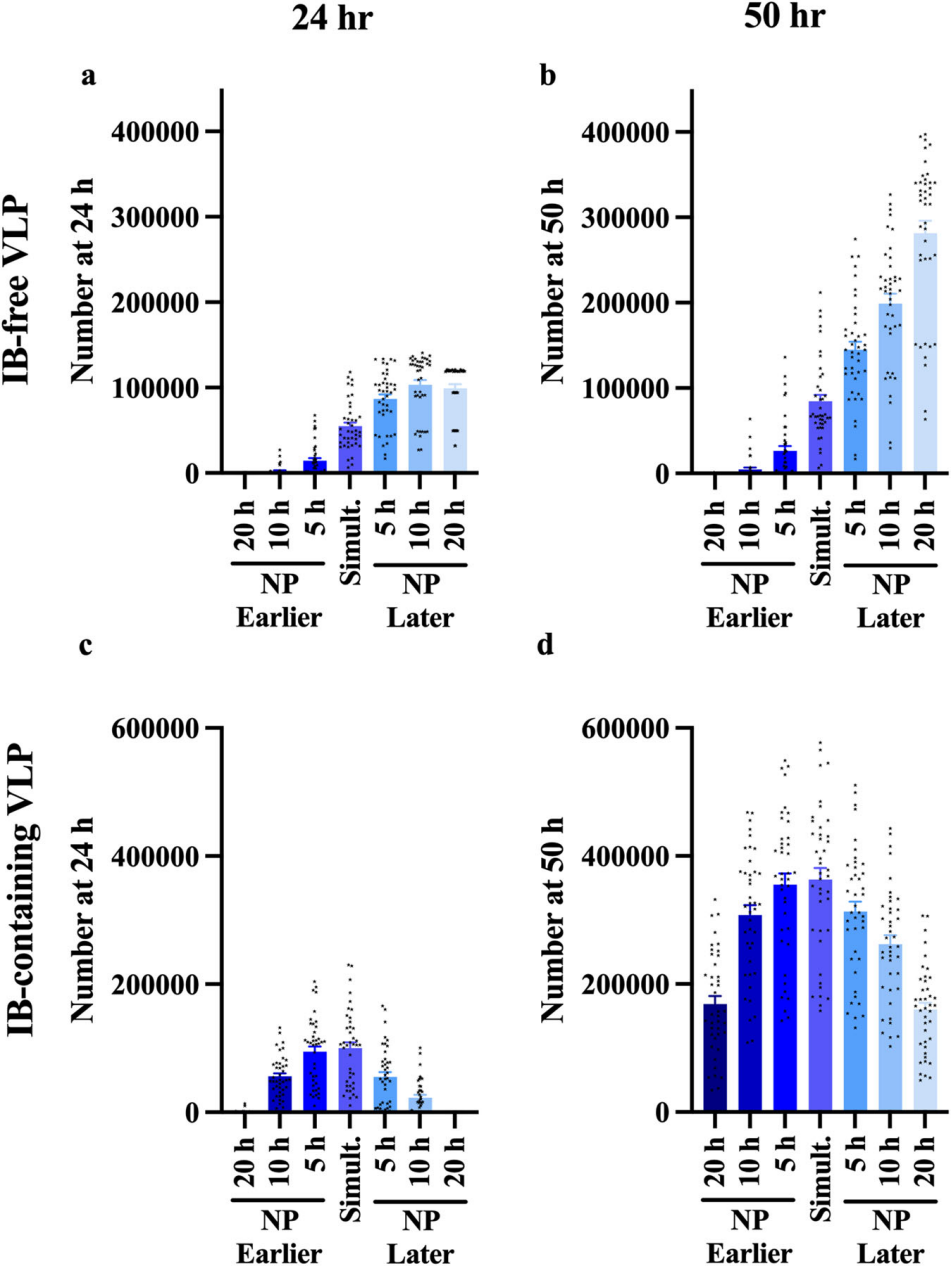
Simulation-predicted influence of NP/VP40 expression time on VLP production. **a**, **b** IB-free VLP production increases as NP expression time (relative to VP40) becomes later. **c**, **d** IB-containing VLP production decreases as the expression time difference between NP and VP40 becomes larger. The highest IB-containing VLP production appears when NP expression time is between 0 and 5 h earlier than VP40 expression time. Error bars indicate SEM. 41 out of 50 groups are used for analysis as others have met tolerance problems in ode-solver. Simult.: Simultaneous start of expression of NP and VP40.

Apart from the expression ratio between NP and VP40, we also wanted to determine the impact of the relative timing of expression of NP relative to VP40 on the production of IB-containing VLPs. To do this, we varied the timing of expression of NP relative to VP40 (from NP expressed 20 h earlier than VP40, to 20 h later than VP40). This analysis demonstrates that the later NP is expressed relative to VP40, the more IB-free VLP will be produced (Fig. 8a, b, Supplementary Data 13). IB-containing VLP production reaches a peak when NP and VP40 are co-expressed at the same time, or when NP expression starts 5 h earlier than VP40 expression (Fig. 8c, d, Supplementary Data 13). These results suggests that an optimum amount of nuclear material-containing EBOV would be produced when NP and VP is expressed at the same time, or NP slightly earlier than VP40. This observation is aligned with the genome sequence of EBOV, as NP is closer to the 3’-end compared to VP40^27^.

Thus, both the expression ratio and the relative expression time of NP and VP40 are important for the effective production of IB-containing viral particles. Our findings suggest that an optimum ratio and expression time exists for maximizing production of functional nuclear material-containing viruses, while minimizing the amount of unused viral proteins.

### Fendiline inhibition of VLP production is weakened when NP is co-expressed

**Fig. 9:**
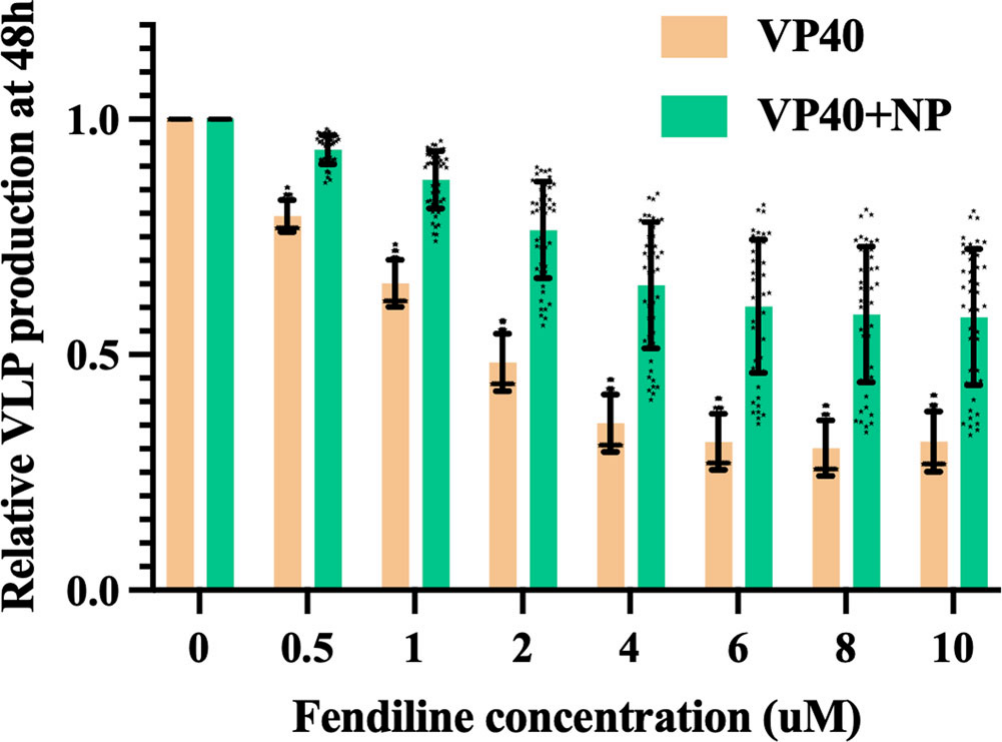
Simulation-predicted inhibition of VLP production by fendiline on VP40-only and NP-VP40 system. While total VLP production is inhibited in both VP40-only and NP-VP40 system by fendiline, the reduction in VLP is smaller in NP-VP40 system. Error bars indicate SD.

We have previously evaluated the ability of fendiline to inhibit EBOV VLP production when VP40 is expressed by itself^25^. Fendiline is known to lower the levels of PS in the host cell membrane, and our work showed that these lower PS levels can lower VP40 association with the membrane, and hamper VLP assembly and budding. Here we investigated if the presence of NP would affect how fendiline-driven PS reduction impacts VLP production. Based on its mechanism of action, fendiline treatment is implemented in the model by modification of the PS cell membrane association rate constant (*k*_5_) (*k*_5Fendiline,Simulation_ in Supplementary Information Table S1). This fendiline concentration-dependent modification was determined through model calibration in previous work^25^. Our results indicate that both IB-free and IB-containing VLP production decreases as fendiline concentration increases (Supplementary Information Fig. S4, Supplementary Data 14). This indicates that fendiline can be effective at suppressing VLP production when NP is co-expressed with VP40. However, if we compare the percentage in VLP between NP co-expression and VP40-only conditions, we find that the inhibition of VLP production by fendiline is weakened in the NP-VP40 system (Fig. 9, Supplementary Data 15). Considering our recent findings^25^ that fendiline is less effective when the VLP budding rate is high, we believe this is caused by the higher budding rate of IB-containing VLPs. Since VLP production can be increased by addition of multiple EBOV proteins^13^, fendiline treatment efficiency may be lower in authentic EBOV infection than our predictions for these simplified VLPs with VP40 and NP only. This is also aligned with experimental findings that fendiline is less effective against live EBOV than VP40 VLP under the same fendiline concentration^28^. Thus, a co-treatment targeting the budding process of EBOV may be important to rescue the efficiency of fendiline, as also suggested in our recent work^25^.

## Discussion

NP is an important viral protein in the EBOV life cycle. In this study, we have incorporated NP into our existing ODE-based VP40 assembly and budding model by a two-stage interaction mechanism and explored the impact of NP on VLP production through computational methods.

A recent study found that the interaction between VP40 and NP can happen in both the cytoplasm and the membrane^17^. Thus, the cytoplasmic IB-bound VP40s and cell membrane-bound VP40s are two different pools. The former is associated with NP in the cytoplasm, and the latter will move to cell membrane, where it recruits IBs and serves as building block for filaments. By explicitly incorporating this two-stage interaction mechanism between NP and VP40, we have successfully replicated experimental observations through our model. Though a previous study indicated no interaction between VP40 and NP in the cytoplasm^22^, the study was conducted in Marburg virus (MARV) instead of EBOV, and NC was used instead of NP alone as in the current study. The difference in species and the discrepancy between NC and IB may explain the observed differences in NP-VP40 interaction. Thus, our model predictions support the two-stage interaction between NP and VP40, and we can further explore a system without cytoplasmic NP-VP40 interaction to assess potentially different mechanisms between different filoviruses.

While a previous study found that VLP production can be increased by co-expression of NP and VP40^13^, our simulations further suggest that this enhancement of VLP production may be due to the stabilization of growing VP40 filaments and increase in VLP budding rate through IB association. These influences will increase VLP budding in general, while reducing the production of IB-free VLPs, which is aligned with experimental observations^13,17^. However, our results also indicate that too much NP may inhibit VLP production by depleting membrane bound VP40 dimers in two ways: (a) high levels of cytoplasmic NP will bind more VP40 and trap them in the cytoplasm; and (b) the stabilized IB-containing VP40 filaments are less likely to release VP40 dimers thereby sequestering membrane-associate VP40 in immature filaments. The combined effect^17^ is that there exists an optimal ratio of NP/VP40 where IB-containing VLPs is maximized. Since we currently lack experimental data on how many VP40s will interact with NPs in the cytoplasm, we are not able to determine the precise ratio of NP/VP40 where NP will start inhibiting VLP production.

On the mRNA level, NP shows a similar level of transcription compared to VP40^29,30^. On the other hand, the size of NP (739 aa) is more than twice that of VP40 (326 aa), so it could be inferred that VP40 translation time should be faster while not considering the difference in amino acid elongation rate caused by codons. Thus, VP40 may be more abundant than NP in EBOV infected cells. Further, since NP is at the 3’ of EBOV genome, the expression should be earlier than VP40. mRNA detection also shows that the level of NP transcription decreases through time^29^. Our simulation indicates that these experimentally observed expression patterns of NP/VP40 timing (slightly earlier expression of NP) and ratio (higher levels of VP40) are beneficial to both the production of functional EBOV particles and the suppression of non-functional EBOV without genetic material. These results suggest that the expression profile of individual EBOV proteins may play a critical role in its life cycle, and thus can be a potential treatment target. Currently, no RNA-based therapy has been approved by the FDA in treating EBOV infection, as all approved treatments of EBOV are antibody-based^5,6^. However, RNA interference (RNAi) has been proposed for viral infection treatment for many years and is considered an efficient means of disruption^31^. Further, there are already experimental therapy using small interfering RNA (siRNA) therapy targeting multiple EBOV proteins^32,33^, but efficacy remains unclear^34^. The difficulty lies in accurate delivery to target cells^31,34,35^, and the efficiency may be affected by application time^34^. However, RNA delivery technology has been greatly advanced recently^36,37^, characterized by the recent mRNA vaccines approved for COVID^38,39^. Our previous work also showed the ability of computational models to assist evaluation of treatment timing^25^. Taken together, our results further suggest that an RNA-based therapy which can disrupt the normal relative expression abundance of EBOV proteins over time could impact infectious virus production.

Our results highlight the power of simplified in vitro and in silico models to disentangle complex protein-protein interaction network structures and dynamics. However, from the fendiline results in this study, we conclude that the interaction between various EBOV proteins can influence treatment efficiency. Thus, our findings also caution against extrapolating drug target conclusions made in simplified in vitro or in silico systems that only consider one viral protein. It will be vital to build the full EBOV infection model for making more accurate efficiency predictions.

Current model limitations include that we do not currently account for viral entry, transcription, replication or the five other EBOV proteins. For the NP-VP40 model, we lack the knowledge of what part of NPs can interact with and with how many VP40s on IBs in cytoplasm, and we currently do not account for the possible interaction between IBs and higher order VP40 oligomers on the host cells membrane. For NP and VP40 assembly processes, the size of oligomers is currently set at fixed values. However, the choice of these parameter values does not affect our qualitative conclusions and would only alter the maxima (but not the shape or dynamics) of IB and VLP size distributions. Nonetheless, our NP-VP40 model successfully reproduces current experimental results, makes important predictions, and provides valuable directions for future experiments.

In summary, our results show how EBOV NP affects viral assembly by influencing filament stability and budding rate constants; how the timing and proportion of NP vs. VP40 expression from the viral genome is potentially optimized for maximal functional virion production; and how viral protein interactions impact drug efficacy. Thus, this work moves the field forward in our understanding of EBOV assembly dynamics; and brings us one step closer a full EBOV infection model, which can be used for in silico treatment trials.

## Methods

### ODE-based model construction

Our model incorporates NP dynamics into our existing model of VP40 assembly and budding^24,25^. Eqs (1)-(2), (8), (10)-(15) and Fig. 1 (right side) describe VP40 assembly and VLP budding dynamics. These equations were derived in previous work^24,25^ based on experimentally identified mechanisms, as well as calibration to experimental data. This previous work illustrated our models’ ability to reproduce key features of experimental data for VP40 expressed alone. Here, we add NP dynamics (Eqs. (3)-(7), (9), (16)-(27), Fig. 1, left side) to our existing equations describing VP40, to computationally represent both NP and VP40 dynamics in mammalian cells expressing both EBOV proteins simultaneously. Evidence and rationale for the NP components in our model are described below, and the combined VP40-NP model structure is summarized in Fig. 1.

In our combined VP40-NP model, VP40 is produced (r_1_), assembled to dimer (*k*_1_, *k*_1_′) and bound to cell membrane (*k*_2_, *k*_2_′). The membrane-bound VP40 dimer can oligomerize to IB-free filaments (*k*_3_, *k*_3,1_′, *k*_3,2_′) and bud in the form of IB-free VLPs (*k*_4_)^24,25^. In this work, we incorporate NP, which is produced (*r*_3_) and assembled into IBs (*k*_6_, *k*_6,1_′, *k*_6,2_′) in the cell cytoplasm^26,40^, and then bound to VP40 dimer at the plasma membrane (*k*_8_, *k*_8_′). We chose this implementation since experimental evidence has not shown whether IB binds to VP40 membrane dimer or higher oligomers, and we make a simplifying assumption that excludes the interaction between IB and higher VP40 oligomers. Cytoplasmic NPs also have the ability to incorporate cytoplasmic VP40 dimers (*k*_7_, *k*_7_′). The ability of IBs to bind to membrane VP40 depends on the cytoplasmic VP40s attached to it^17^. Finally, membrane VP40 can oligomerize into IB-containing filaments (*k*_9_, *k*_9,1_′, *k*_9,2_′), and be released in the form of IB-containing VLPs (*k*_10_, Fig. 1). We assume that the ratio between nucleation and elongation dissociation rate constants in the IB-containing VP40 filament (*k*_9,2_′ to *k*_9,1_′) is identical to that in IB-free VP40 filament (*k*_3,2_′ to *k*_3,1_′). The structure of the equations assumes mass action kinetics, and the assembly of large size oligomers is realized by adding the corresponding molecules one by one.

ODEs for the model are shown in Eqs. (1)–(24).

*A*: VP40 monomer in cytoplasm (nM).

1 $$\frac{{dA}}{{dt}}={r}_{1}-2{k}_{1}{A}^{2}+2{k}_{1}^{{\prime} }B-{d}_{1}A$$

*B*: VP40 dimer in cytoplasm (nM).

2 $$\frac{{dB}}{{dt}}=\frac{3\left({k}_{1}{A}^{2}-{k}_{1}^{{\prime} }B-{k}_{2}{{BD}}^{{\prime} }+{k}_{2}^{{\prime} }{E}_{1}\right)}{R}-{k}_{7}\left({\sum}_{i=1}^{{n}_{1}}i{C}_{i}-J\right)B+{k}_{7}^{{\prime} }J$$

*C*_*i*_: Developing cytoplasm IB consists of i NPs (nM).

*i*: Number of NPs in cytoplasm IB.

*z*_1_: Size of IB where the reverse rate constant change from *k*_6,1_′to *k*_6,2_′ from nucleation to elongation

*n*_1_: Number of NPs contained in a mature IB. *n*_1_ = 800 in our model.

3 $$\frac{d{C}_{1}}{{dt}}={r}_{3}-2{k}_{6}{C}_{1}^{2}+2{k}_{6,1}^{{\prime} }{C}_{2}-{k}_{6}{C}_{1}{\sum}_{i=2}^{{n}_{1}-1}{C}_{i}+{k}_{6,1}^{{\prime} }{\sum}_{i=3}^{{n}_{1}}{C}_{i}-{d}_{1}{C}_{1}$$

4 $$\frac{d{C}_{i}}{{dt}}={k}_{6}{C}_{1}{C}_{i-1}-{k}_{6,1}^{{\prime} }{C}_{i}-{k}_{6}{C}_{1}{C}_{i}+{k}_{6,1}^{{\prime} }{C}_{i+1}\,(1 < i < {z}_{1})$$

5 $$\frac{d{C}_{{z}_{1}}}{{dt}}={k}_{6}{C}_{1}{C}_{{z}_{1}-1}-{k}_{6,1}^{{\prime} }{C}_{{z}_{1}}-{k}_{6}{C}_{1}{C}_{{z}_{1}}+{k}_{6,2}^{{\prime} }{C}_{{z}_{1}+1}$$

6 $$\frac{d{C}_{i}}{{dt}}={k}_{6}{C}_{1}{C}_{i-1}-{k}_{6,2}^{{\prime} }{C}_{i}-{k}_{6}{C}_{1}{C}_{i}+{k}_{6,2}^{{\prime} }{C}_{i+1}\,({z}_{1} < i < {n}_{1})$$

7 $$\frac{d{C}_{{n}_{1}}}{{dt}}={k}_{6}{C}_{1}{C}_{{n}_{1}-1}-{k}_{6,2}^{{\prime} }{C}_{{n}_{1}}+\frac{3\left({k}_{8}^{{\prime} }{I}_{1}-{k}_{8}{C}_{{n}_{1}}{F}_{1}\right)}{R}$$

*D*: Plasma membrane phosphatidylserine (nmol/dm^2^).

8 $$\frac{{dD}}{{dt}}=\frac{{k}_{5}{GR}}{3}-{k}_{5}^{{\prime} }D-{k}_{2}{{BD}}^{* }+{k}_{2}^{{\prime} }{E}_{1}$$

*E*_*j*_: Developing IB-free VP40 filament consists of *j* VP40 dimers (nmol/dm^2^).

*j*: Number of dimers in developing filament.

*z*_2_: Size of VP40 filament where the reverse rate constant changes from *k*_3,1_′to *k*_3,2_′ (from nucleation to elongation).

*n*_2_: Number of dimers in a mature filament. *n*_2_ = 2310 in our model.

9 $$\frac{d{E}_{1}}{{dt}}= 	\, {k}_{2}{{BD}}^{{\prime} }-{k}_{2}^{{\prime} }{E}_{1}-2{k}_{3}{{E}_{1}}^{2}-{k}_{3}{E}_{1}{\sum}_{j=2}^{{n}_{2}-1}{E}_{j}+2{k}_{3,1}^{{\prime} }{E}_{2}+{k}_{3,1}^{{\prime} }{\sum}_{j=3}^{{z}_{2}}{E}_{j}\\ 	 +{k}_{3,2}^{{\prime} }{\sum}_{j={z}_{2}+1}^{{n}_{2}}{E}_{j}+{k}_{8}^{{\prime} }{H}_{1}-{k}_{8}{C}_{{n}_{1}}{E}_{1}-{k}_{9}{E}_{1}{\sum}_{j=1}^{{n}_{2}-1}{H}_{j}\\ 	 +{k}_{9,1}^{{\prime} }{\sum}_{j=2}^{{z}_{2}}{H}_{j}+{k}_{9,2}^{{\prime} }{\sum}_{j={z}_{2}+1}^{{n}_{2}}{H}_{j}$$

10 $$\frac{d{E}_{i}}{{dt}}={k}_{3}{E}_{1}{E}_{j-1}-{k}_{3,1}^{{\prime} }{E}_{j}-{k}_{3}{E}_{1}{E}_{j}+{k}_{3,2}^{{\prime} }{E}_{j+1}\,(1 < j < {z}_{2})$$

11 $$\frac{d{E}_{{z}_{2}}}{{dt}}={k}_{3}{E}_{1}{E}_{{z}_{2}-1}-{k}_{3,1}^{{\prime} }{E}_{{z}_{2}}-{k}_{3}{E}_{1}{E}_{{z}_{2}}+{k}_{3,2}^{{\prime} }{E}_{{z}_{2}+1}$$

12 $$\frac{d{E}_{i}}{{dt}}={k}_{3}{E}_{1}{E}_{j-1}-{k}_{3,2}^{{\prime} }{E}_{j}-{k}_{3}{E}_{1}{E}_{j}+{k}_{3,2}^{{\prime} }{E}_{j+1}\,({z}_{2} < j < {n}_{2})$$

13 $$\frac{d{E}_{{n}_{2}}}{{dt}}={k}_{3}{E}_{1}{E}_{{n}_{2}-1}-{k}_{3,2}^{{\prime} }{E}_{{n}_{2}}-{k}_{4}{E}_{{n}_{2}}$$

*F*: Budded IB-free VLP (nmol/dm^2^).

14 $$\frac{{{{{{\rm{dF}}}}}}}{{{{{{\rm{dt}}}}}}}={{{{{{\rm{k}}}}}}}_{4}{{{{{{\rm{E}}}}}}}_{{{{{{{\rm{n}}}}}}}_{2}}$$

*G*: Cytoplasmic phosphatidylserine (nM).

15 $$\frac{{dG}}{{dt}}={r}_{2}-{k}_{5}G+\frac{3{k}_{5}^{{\prime} }D}{R}-{d}_{2}G$$

*H*_*j*_: Developing IB-containing VP40 filament consists of j VP40 dimers (nmol/dm^2^).

16 $$\frac{d{H}_{1}}{{dt}}={k}_{8}{C}_{{n}_{1}}{E}_{1}-{k}_{8}^{{\prime} }{H}_{1}-{k}_{9}{H}_{1}{E}_{1}+{k}_{9,1}^{{\prime} }{H}_{2}$$

17 $$\frac{d{H}_{i}}{{dt}}={k}_{9}{E}_{1}{H}_{j-1}-{k}_{9,1}^{{\prime} }{H}_{j}-{k}_{9}{E}_{1}{H}_{j}+{k}_{9,2}^{{\prime} }{H}_{j+1}\,(1 < j < {z}_{2})$$

18 $$\frac{{{{{{\rm{d}}}}}}{{{{{{\rm{H}}}}}}}_{{{{{{{\rm{z}}}}}}}_{2}}}{{{{{{\rm{dt}}}}}}}={{{{{{\rm{k}}}}}}}_{9}{{{{{{\rm{E}}}}}}}_{1}{{{{{{\rm{H}}}}}}}_{{{{{{{\rm{z}}}}}}}_{2}-1}-{{{{{{\rm{k}}}}}}}_{9,2}^{{\prime} }{{{{{{\rm{H}}}}}}}_{{{{{{{\rm{z}}}}}}}_{2}}-{{{{{{\rm{k}}}}}}}_{9}{{{{{{\rm{E}}}}}}}_{1}{{{{{{\rm{H}}}}}}}_{{{{{{{\rm{z}}}}}}}_{2}}+{{{{{{\rm{k}}}}}}}_{9,2}^{{\prime} }{{{{{{\rm{H}}}}}}}_{{{{{{{\rm{z}}}}}}}_{2}+1}$$

19 $$\frac{d{H}_{i}}{{dt}}={k}_{9}{E}_{1}{H}_{j-1}-{k}_{9,2}^{{\prime} }{H}_{j}-{k}_{9}{E}_{1}{H}_{j}+{k}_{9,2}^{{\prime} }{H}_{j+1}\,({z}_{2} < j < {n}_{2})$$

20 $$\frac{d{H}_{{n}_{2}}}{{dt}}={k}_{9}{E}_{1}{H}_{{n}_{2}-1}-{k}_{9,2}^{{\prime} }{H}_{{n}_{2}}-{k}_{4}{H}_{{n}_{2}}$$

*I*: Budded IB-containing VLP (nmol/dm^2^).

21 $$\frac{{dI}}{{dt}}={k}_{10}{H}_{{n}_{2}}$$

*J*: Cytoplasmic VP40 dimer trapped in cytoplasm IB (nM).

22 $$\frac{{dJ}}{{dt}}={k}_{7}\left({\sum}_{i=1}^{{n}_{1}}i{C}_{i}-J\right)B-\,{k}_{7}^{{\prime} }J-\frac{3\left({k}_{8}{C}_{{n}_{1}}{E}_{1}{P}_{1}+{k}_{8}^{{\prime} }{H}_{1}{P}_{2}\right)}{R}$$

*K*: Trapped VP40 dimer in plasma membrane IB (nmol/dm^2^).

23 $$\frac{{dK}}{{dt}}={k}_{8}{C}_{{n}_{1}}{E}_{1}{P}_{1}-\left({k}_{8}^{{\prime} }{H}_{1}+{k}_{10}{H}_{{n}_{2}}\right){P}_{2}$$

*L*: Trapped VP40 dimer in budded IB-containing VLP (nmol/dm^2^).

24 $$\frac{{dL}}{{dt}}={k}_{10}{H}_{{n}_{2}}{P}_{2}$$

Useful ratios P_1_-P_3_ are defined in Eqs. (25)–(27).

*P*_1_: Portion of cytoplasmic NP bound by VP40 dimer.

25 $${P}_{1}=\frac{J}{{\sum}_{i=1}^{{n}_{1}}i{C}_{i}}$$

*P*_2_: Portion of plasma membrane NP bound by VP40 dimer.

26 $${P}_{2}=\frac{K}{{n}_{1}{\sum }_{i=1}^{{n}_{2}}{H}_{i}}$$

*P*_3_: Portion of budded NP bound by VP40 dimer.

27 $${P}_{3}=\frac{L}{{n}_{1}I}$$

Initial conditions:

$$A\left(0\right)=0$$

$$B\left(0\right)=0$$

$${C}_{i}\left(0\right)=0$$

$${C}_{i}\left(0\right)=0\,\left(1\le i\le {n}_{1}\right)$$

$$D\left(0\right)=16.75$$

$${E}_{j}\left(0\right)=0\,\left(1\le j\le {n}_{2}\right)$$

$$F\left(0\right)=0$$

$$G\left(0\right)=1.07\times {10}^{5}$$

$${H}_{j}\left(0\right)=0\,\left(1\le j\le {n}_{2}\right)$$

$$I\left(0\right)=0$$

$$J\left(0\right)=0$$

$$K\left(0\right)=0$$

$$L\left(0\right)=0$$

The calculation of D^*^ is shown in Eq. (28). The deduction of the equation is detailed in our prior work^25^.

D^*^: Plasma membrane Phosphatidylserine available to interact with cytoplasmic VP40 dimer (nmol/dm^2^).

28 $${D}^{* }=1.43\times {10}^{-3}\times \frac{D}{1.07\times {10}^{5}}$$

### Influence of IB-bound VP40 on IB cell membrane association

Previous work has shown that the interaction of NP NTD with cytoplasmic VP40 can cause a conformational change in the NP CTD, which is critical for the recruitment of IB to cell membrane^17^. We reflect this mechanism in our model, by having the IB membrane association rate constant (*k*_8_) positively impacted by the portion of its NP occupied by cytoplasmic VP40 dimer. The influence is described in Eq. (29).

29 $${k}_{8}=\frac{{k}_{8{{{{{\rm{\_}}}}}}0}}{\left(1-{y}_{1}\times \left(1-\exp \left(-\left(\frac{J}{{\sum }_{i=1}^{{n}_{1}}i{C}_{i}}-0.5\right)\times {y}_{2}\right)\right)\right)}$$

Values of *y*_1_ and *y*_2_ are listed in Supplementary Information Table S1.

*k*_8_0_: Calibrated IB plasma membrane association rate constant without considering the effect of attached cytoplasmic VP40 (Supplementary Information Table S1).

### Experimental data

Three groups of data from two NP-VP40 experimental studies are used to calibrate our model:

- NP VLP production ratio: defined as the ratio of VLP production with NP co-expression relative to VLP production with expression of VP40 alone at 24 to 30 h^13^. It was measured to be 3.6 (standard deviation (SD): 1.961) in a wild-type NP + VP40 experiment.
- CTD-mutant NP membrane VP40 ratio: defined as the ratio of cell membrane VP40 number relative to cytoplasmic VP40 number in both WT and CTD-mutant NP co-expression at 24 h^17^. It was measured to be 6.33 (SD: 1.55) and 0.64 (SD: 0.14) in the WT and mutant respectively.
- CTD-mutant NP (L692A, P697A, P698A, W699A, which have CTD mutations and are compromised in binding to the cell membrane) VLP production ratio: defined as the ratio of VLP production with wild-type (WT) NP co-expression with VP40 relative to CTD-mutant NP co-expression with VP40 at 36 h^17^. It was measured to be 0.31 (SD: 0.13).

CTD-mutant NP data are combined together in our calibration since they are all mutated in the CTD core and are also analyzed collectively in the experimental work^17^. We reflect this mutation mathematically by letting k_8_ = 0 for these mutants.

As qualitative validation of the NP-VP40 model, we evaluate the fitted parameters by their ability to reproduce the observed dynamics that when NP is expressed by itself, the average IB size increases over time; and that the IBs have a bimodal size distribution, with the majority of IBs being either very large or very small at latter time^26^.

### Calibration and parameter estimation

NP-related parameters are sampled through Latin hypercube sampling (LHS) in a wide range, while VP40-only parameters are randomly sampled from the 75 “As2” simulation groups in our last study^25^. Parameters that are varied for calibration in this work are marked in Supplementary Information Table S1. The size of NP IBs and VLPs (*n*_*1*_ and *n*_*2*_) are calculated from the experimentally determined size of VP40 filaments and the ratio between VP40 and NP^25,41^. The choice of these parameter values does not affect our qualitative conclusions and would only alter the maxima (but not the shape or dynamics) of IB and VLP size distributions. The agreement of model predictions with experimental data is calculated by a cost function as described in our last study and shown in Eq. (30). All predictions at a certain time point are calculated from the average value within ±2 h as in our previous work^25^.

30 $${{{{{\rm{cost}}}}}}=\frac{{\sum }_{{{{{{\rm{q}}}}}}=1}^{{{{{{\rm{N}}}}}}}\left({\sum }_{{{{{{\rm{j}}}}}}=1}^{{{{{{\rm{M}}}}}}\left({{{{{\rm{q}}}}}}\right)}\left(\frac{\frac{\max \left({{{{{{\rm{p}}}}}}}_{{{{{{\rm{j}}}}}},{{{{{\rm{q}}}}}}},{{{{{{\rm{e}}}}}}}_{{{{{{\rm{j}}}}}},{{{{{\rm{q}}}}}}}\right)}{\min \left({{{{{{\rm{p}}}}}}}_{{{{{{\rm{j}}}}}},{{{{{\rm{q}}}}}}},{{{{{{\rm{e}}}}}}}_{{{{{{\rm{j}}}}}},{{{{{\rm{q}}}}}}}\right)}-1}{{{{{{\rm{M}}}}}}\left({{{{{\rm{q}}}}}}\right)}\right)\right)\,}{{{{{{\rm{N}}}}}}}$$

N: Number of different data types

M(q): Number of data in the qth data type

e^j,q^: jth experiment data in the qth data type

p^j,q^: jth model prediction in the qth data type

Calibration is performed in an iterative manner. In each iteration, 2500 initial guesses are sampled with LHS. The top 50 parameter sets with the lowest costs are used for determination of the parameter ranges of next iteration. Since experimental studies showed that IB-containing VLPs are dominant compared to IB-free VLPs at 36 h post-transfection^17^, we also use this feature to filter parameter sets for further analysis starting from the 3rd iteration (such cases are very limited in iterations 1–2, Supplementary Data 1). The ranges of calibrated parameters in each round are shown in Supplementary Data 2, and the distribution of parameter values in the 50 selected parameter sets is included in Supplementary Information Fig. S5. Calibration is considered complete after six iterations due to an increase in cost in the seventh iteration compared to the sixth. Some parameter sets have P_1_-P_3_ higher than 1 at some time points due to numerical errors and are excluded from further analysis and simulation. The top 50 best fit parameter sets from the rest of the sixth iteration samples are selected for further analysis (Supplementary Data 3). Given the small sample sizes of our experimental datasets, they do not necessarily reflect the true distribution of the system. Therefore, in evaluating multiple parameter sets we are not attempting to reproduce the true variability and distribution of the underlying biological system, but instead trying to capture the range of possible outcomes.

### Simulation

Three kinds of simulations are performed: local sensitivity analysis, variable expression time, and fendiline treatment. In local sensitivity analysis, selected parameters are changed from 0.1× to 10× of their original values, while other parameters remain the same. These local sensitivity analyses allows us to evaluate how the impact of NP on different steps in VP40 assembly and budding process can influence VLP production, as well as the importance of the NP/VP40 expression ratio. In variable expression time simulations, the relative monomer production starting time of NP ranges from 20 h earlier than VP40 to 20 h later. The variable expression time simulations will enable us to assess if the relative expression time of NP/VP40 is important in EBOV infection. In fendiline treatment, 0.5 µM–10 µM of fendiline is simulated in the NP-VP40 system to test how disruption of phosphatidylserine by fendiline affects VLPs when NP is co-expressed with VP40.

### Statistics and reproducibility

All data and model code necessary to reproduce results are available in either Supplementary Data or as described in the code availability statement. Sample size is 50 in all simulations, unless stated otherwise in figure captions. Input parameters are different for each replicate of our simulations. Sample sizes for experimental data is given in figure captions.

For comparing ratios to baseline values (Fig. 2a, c) a one sample, two-tailed t-test was performed. For comparing mutant to WT values (Fig. 2b) an unpaired, two-tailed t-test was performed. Significance level of α = 0.05 was used for all tests.

### Software application

The ODE model is implemented in Matlab R2022a. Solver “ode23t” is used for solving ODEs, with the analytical Jacobian Matrix provided, and using the “NonNegative” setting to avoid negative values. All result figures and statistical analysis are created with Graphpad Prism.

### Reporting summary

Further information on research design is available in the Nature Portfolio Reporting Summary linked to this article.

### Supplementary information

Supplementary Information

Description of Additional Supplementary Files

Supplemental Data 1–3

Supplemental Data 4-6

Supplemental Data 7-8

Supplemental Data 9-12

Supplemental Data 13

Supplemental Data 14-15

Reporting Summary

## Data Availability

The source data behind the graphs in the paper can be found in Supplementary Data 1-15.

## Code availability

Model code is available through Zenodo at zenodo.org/records/8188145 with DOI 10.5281/zenodo.8188145^42^.
