# Supplementary material for: Impact of Ebola virus nucleoprotein on VP40 virus-like particle production: a computational approach
Source: Commun Biol. 2024 May 25;7:634. doi: 10.1038/s42003-024-06300-8 (PMC11128010; doi:10.1038/s42003-024-06300-8)
Supplement: Supplementary file 1 — Supplementary Information [file 42003_2024_6300_MOESM1_ESM.docx]

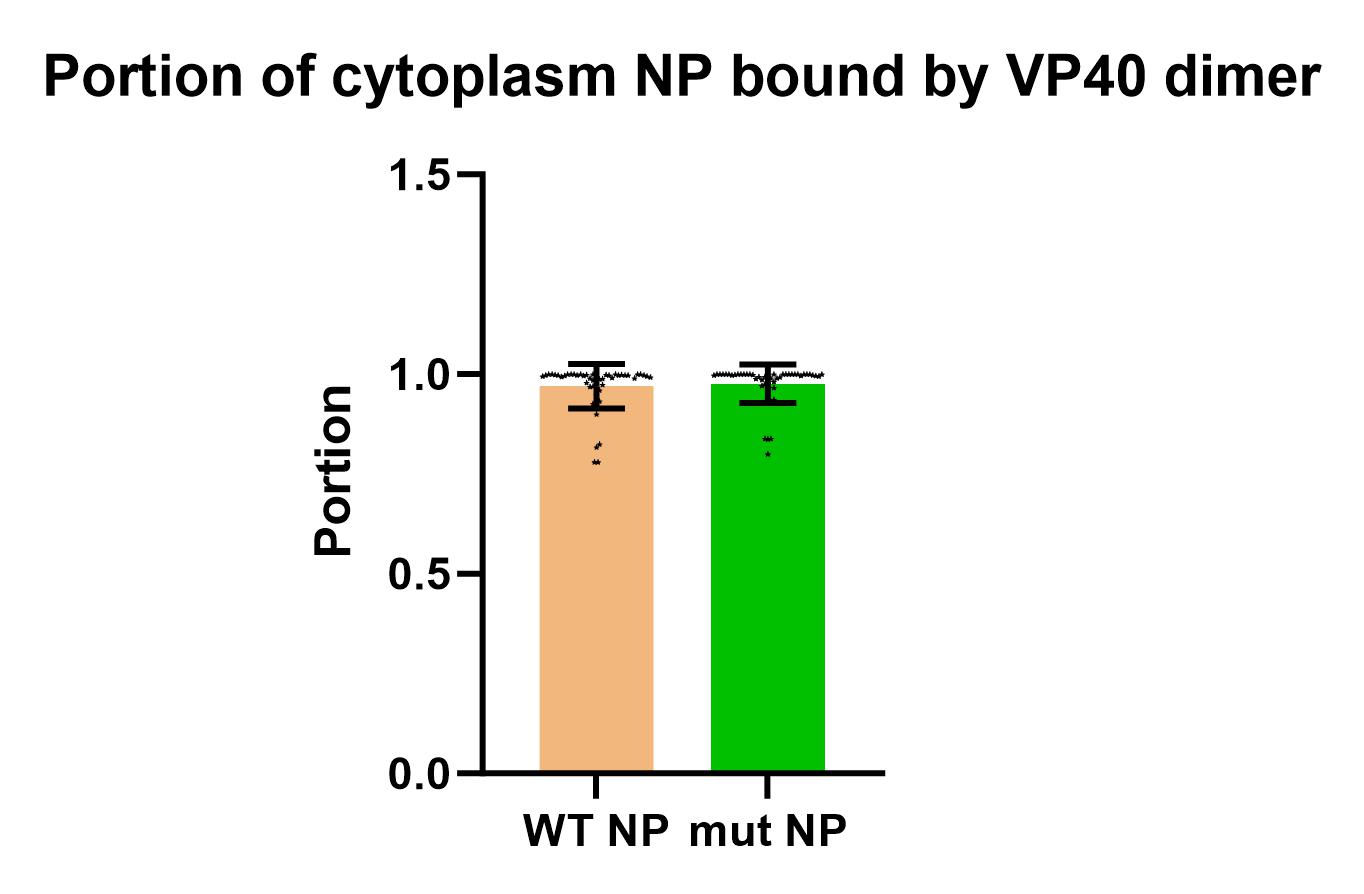


**Supplementary Information Figure S1. Values of P_1_ at 30h in selected groups.** *P*_1_ is slightly increased when NP is mutant, indicating stronger binding to cytoplasmic VP40. Error bars indicate SD.


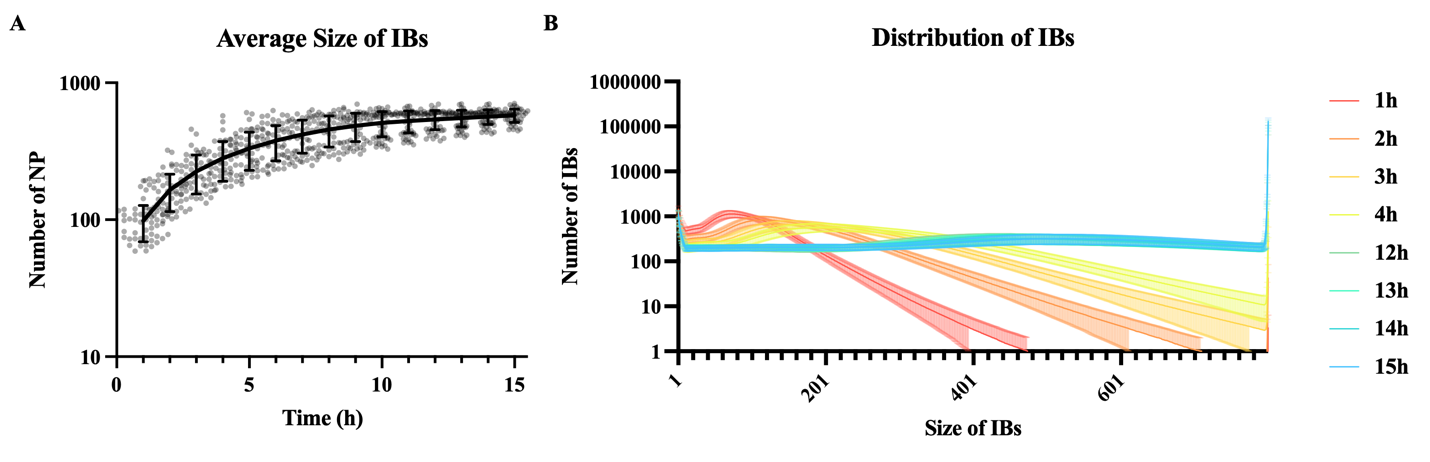


**Supplementary Information Figure S2. The size of IBs in a NP-only system.** (a) Average size of IBs increases over time for 1-15h. . Error bars indicate SD, points show individual points with random x-direction shift for visibility. (b) Distribution of IBs becomes more and more binary at later timepoints. Error bars indicate 95% CI. Note that the average IB size plateaus near the maximum size (*n*_1_ = 800) as the majority of IBs are at or near the maximum size. Nonetheless, the bimodal size distribution remains stable beyond 12 hrs.

**Supplementary Information Figure S3. Influence of NP production rate on system dynamics.** (a) Cytoplasmic IB-bound VP40 increases as r_3_ increases from 0.1× to 10×. (b) Cell membrane VP40 increases when r_3_ is either very small or large. (c) Total VLP production decreases when r_3_ is either very small or large, especially very large. (d) Cell membrane VP40 dimer decreases when r_3_ increases from 0.1× to 10×. (e) Cell membrane NP increases when r_3_ increases from 0.1× to 10×. Bars show means, error bars indicate SEM at 24 hrs (left panels) and 50 hrs (right panels). 31 out of 50 groups are used for analysis as others have met tolerance problems in ode-solver.


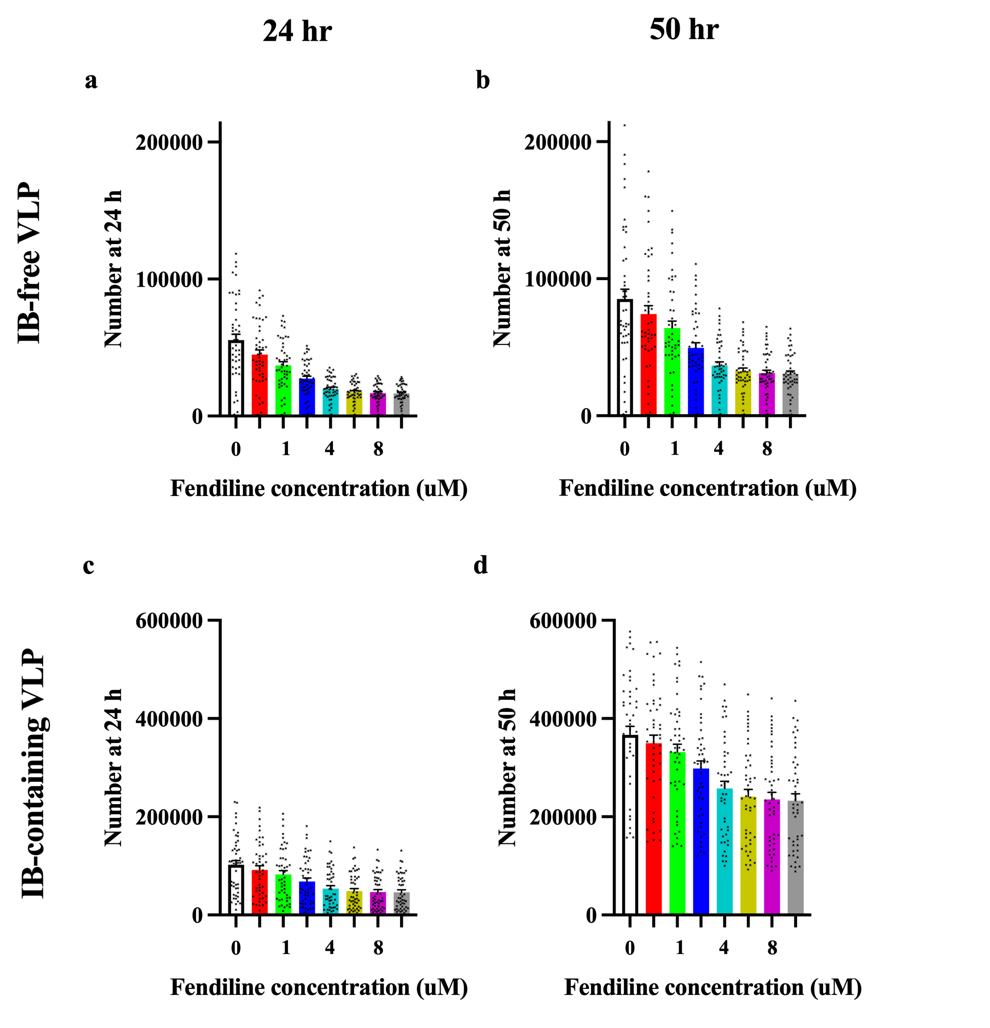


**Supplementary Information Figure S4. Influence of fendiline on VLP production.** Both VLPs without IBs (a, b) and IB-containing VLPs (c, d) are inhibited by fendiline. The inhibition increases with fendiline concentration. Error bars indicate SEM. Panels a and c show 24 hr values, and panels b and d show 50 hr values.

**Supplementary Information Figure S5. Calibrated parameter distributions.** The final 50 parameter set distributions are shown. Box bottom and top represent the min and max values respectively. Center lines represent the median, and points represent individual values.

**Supplementary Information Table S1. Model parameters.**

| Parameter | Value | Unit | Description |
| --- | --- | --- | --- |
| *k*_1_ | Calibrated in (1) ^*^ | 1/(nM s) | VP40 dimer association rate constant |
| *KD*_1_ | 50 (1, 2) | nM | VP40 dimer dissociation constant |
| *k*_1_′ | *k*_1_×*KD*_1_ (1, 2) | 1/s | VP40 dimer dissociation rate constant |
| *k*_2_ | *k*_2_′/ *KD*_2_ (1, 2) | 1/(nM s) | VP40 cell membrane association rate constant |
| *KD*_2_ | 2.18×10^3^/exp(0.112×*PS*) (1) | nM | VP40 cell membrane dissociation constant |
| *k*_2_′ | Calibrated in (1) ^*^ | 1/s | VP40 cell membrane dissociation rate constant |
| *k*_3_ | Calibrated in (1) ^*^ | dm^2^/(nmol s) | IB-free VP40 filament association rate constant |
| *KD*_3,1_ | Calibrated in (1) ^*^ | nmol/dm^2^ | IB-free VP40 filament nucleation dissociation constant |
| *k*_3,1_′ | *k*_3_×*KD*_3,1_/(1-0.5×(1-exp((*PS*-20)×0.1))) (1) | 1/ s | IB-free VP40 filament nucleation dissociation rate constant |
| *KD*_3,2_ | Calibrated in (1) ^*^ | nmol/dm^2^ | IB-free VP40 filament elongation dissociation constant |
| *k*_3,2_′ | k_3_×*KD*_3,2_ (1) | 1/ s | IB-free VP40 filament elongation dissociation rate constant |
| *k*_4_0_ | Calibrated in (1) ^*^ | 1/s | IB-free VLP budding rate without PS regulation |
| *k*_4_ | *k*_4_0_ /(1-0.5×(1-exp(-(*PS*-20)×*x*))) (1) | 1/s | IB-free VLP budding rate |
| *k*_5_ | 6×*k*_5_′ (1) | 1/s | PS cell membrane association rate constant |
| *k*_5Fendiline,Simulation_ | (0.364×exp(-0.498×ConcFendiline)-0.364+1)×*k*_5_ (1) ^#^ | 1/s | PS cell membrane association rate constant with fendiline treatment in fendiline simulation |
| *k*_5_′ | Calibrated in (1) ^*^ | 1/s | PS cell membrane dissociation rate constant |
| *k*_6_ | Calibrated | 1/(nM s) | IB association rate constant |
| *KD*_6,1_ | Calibrated | nM | IB nucleation dissociation constant |
| *k*_6,1_′ | *k*_6_×*KD*_6,1_ | 1/s | IB nucleation dissociation rate constant |
| ratio_1_ | Calibrated | 1 | Ratio between IB elongation dissociation constant and IB nucleation dissociation rate constant |
| *KD*_6,2_ | ratio_1_×*KD*_6,1_ | nM | IB elongation dissociation constant |
| *k*_6,2_′ | *k*_6_×*KD*_6,2_ | 1/s | IB elongation dissociation rate constant |
| *k*_7_ | Calibrated | 1/(nM s) | Cytoplasm VP40 dimer-IB association rate constant |
| *KD*_7_ | Calibrated here | nM | Cytoplasm VP40 dimer-IB dissociation constant |
| *k*_7_′ | *k*_7_×*KD*_7_ | 1/s | Cytoplasm VP40 dimer-IB dissociation rate constant |
| *k*_8_0_ | Calibrated | 1/(nM s) | Cytoplasm IB membrane association rate constant without attached VP40 dimer regulation |
| *k*_8_ | *k*_8_0_/((1-*y*_1_×(1*-*exp(-(*J*/(∑(*i*×*C_i_*))-0.5)×*y*_2_)))) (1≤*i*≤*n*_1_) (Eq. 29) | 1/(nM s) | Cytoplasm IB membrane association rate constant with attached VP40 dimer regulation |
| *KD*_8_ | Calibrated | nM | Cytoplasm IB membrane dissociation constant |
| *k*_8_′ | *k*_8_×*KD*_8_ | 1/s | Cytoplasm IB membrane VP40 dimer dissociation rate constant |
| ratio_2_ | Calibrated | 1 | Ratio between IB-containing VP40 filament association rate constant and IB-free VP40 filament association rate constant |
| *k*_9_ | *k*_3_×ratio_2_ | dm^2^/(nmol s) | IB-containing VP40 filament association rate constant |
| ratio_3_ | Calibrated | 1 | Ratio between IB-containing VP40 filament nucleation dissociation constant and IB-free VP40 filament nucleation dissociation constant |
| *KD*_9,1_ | *KD*_3,1_×ratio_3_ | nmol/dm^2^ | IB-containing VP40 filament nucleation dissociation constant |
| *k*_9,1_′ | *k*_9_×*KD*_9,1_/(1-0.5×(1-exp((*PS*-20)×0.1))) | 1/ s | IB-containing VP40 filament nucleation dissociation rate constant |
| *KD*_9,2_ | *KD*_3,2_×ratio_3_ | nmol/dm^2^ | IB-containing VP40 filament elongation dissociation constant |
| *k*_9,2_′ | *k*_9_×*KD*_9,2_ | 1/ s | IB-containing VP40 filament elongation dissociation rate constant |
| ratio_4_ | Calibrated | 1 | Ratio between IB-containing VLP budding rate and IB-free VLP budding rate |
| *k*_10_ | k_4_×ratio_4_ | 1/s | IB-containing VLP budding rate |
| *r*_1_ | Calibrated in (1) ^*^ | nM/s | VP40 production rate |
| *r*_2_0_ | Calibrated in (1) | nM/s | PS production rate without PS regulation |
| *r*_2_ | *r*_2_0_×0.942^11.7^/(0.942^11.7^+(*PS*/20)^11.7^) | nM/s | PS production rate |
| ratio_5_ | Calibrated | 1 | Ratio between NP production rate and VP40 production rate |
| r_3_ | r_1_×ratio_5_ | nM/s | NP production rate |
| *d*_1_ | 2.25×10^-5^ | 1/s | Protein degradation rate |
| *d*_2_ | *r*_2_/*G*(0) | 1/s | PS degradation rate |
| *n*_1_ | 800 (1, 3) | 1 | Size of NP IB |
| *n*_2_ | 2310 (1) | 1 | Size of VP40 filament |
| *R* | 7.82×10^-5^ | dm | Cell radius |
| *x* | Calibrated in (1) | 1 | Manual parameter to control influence of PS on VLP budding rate |
| *y*_1_ | 0.5 | 1 | Manual parameter to control influence of cytoplasmic IB-bound VP40 dimer on IB membrane association rate constant. |
| *y*_2_ | Calibrated | 1 | Manual parameter to control influence of cytoplasmic IB-bound VP40 dimer on IB membrane association rate constant. |
| *z*_1_ | Calibrated | 1 | Size of IB where nucleation changes to elongation |
| *z*_2_ | Calibrated | 1 | Size of VP40 filament where nucleation changes to elongation |

*: Randomly sampled from 75 “As2” parameter sets in our previous work.

#: Only used in fendiline treatment. Fendiline concentration range from 0.5-10µM.

Parameter names are italicized.

**Supplementary Data 1. Summary of calibration result for each round.**

See “Supplementary_Information_calibration.xlsx”

**Supplementary Data 2. Parameter ranges for each round.**

See “Supplementary_Information_calibration.xlsx”

**Supplementary Data 3. Chosen parameter sets for analysis and simulations.**

See “Supplementary_Information_calibration.xlsx”

**Supplementary Data 4. Portion of cytoplasmic NP bound by VP40 in chosen parameter sets.**

See “Supplementary_Information_validation.xlsx”

**Supplementary Data 5. Change of the average IB size in chosen parameter sets.**

See “Supplementary_Information_validation.xlsx”

**Supplementary Data 6. Distribution of IB sizes in chosen parameter sets.**

See “Supplementary_Information_validation.xlsx”

**Supplementary Data 7. VLP production in chosen parameters.**

See “Supplementary_Information_analysis.xlsx”

**Supplementary Data 8. Parameter comparison between NP+VP40 and VP40 system.**

See “Supplementary_Information_analysis.xlsx”

**Supplementary Data 9. VLP production in local sensitivity analysis for NP impact on filament dissociation constant.**

See “Supplementary_Information_local_sensitivity_analsysis.xlsx”

**Supplementary Data 10. VLP production in local sensitivity analysis for NP impact on VLP budding rate constant.**

See “Supplementary_Information_local_sensitivity_analsysis.xlsx”

**Supplementary Data 11. VLP production in local sensitivity analysis for NP/VP40 production ratio.**

See “Supplementary_Information_local_sensitivity_analsysis.xlsx”

**Supplementary Data 12. NP and VP40 profiles in local sensitivity analysis for NP/VP40 production ratio.**

See “Supplementary_Information_local_sensitivity_analsysis.xlsx”

**Supplementary Data 13. VLP production in expression time test.**

See “Supplementary_Information_expression_time_test.xlsx”

**Supplementary Data 14. VLP production in fendiline treatment.**

See “Supplementary_Information_fendiline_treatment.xlsx”

**Supplementary Data 15. VLP reduction at 24 and 48h in fendiline treatment.**

See “Supplementary_Information_fendiline_treatment.xlsx”

**References**

1. Liu, X., Husby, M., Stahelin, R. V., and Pienaar, E. (2024) Evaluation of fendiline treatment in VP40 system with nucleation-elongation process: a computational model of Ebola virus matrix protein assembly. *Microbiol Spectr*. 10.1128/spectrum.03098-23

2. Liu, X., Pappas, E. J., Husby, M. L., Motsa, B. B., Stahelin, R. v., and Pienaar, E. (2022) Mechanisms of phosphatidylserine influence on viral production: A computational model of Ebola virus matrix protein assembly. *Journal of Biological Chemistry*. **298**, 102025

3. Elliott, L. H., Kiley, M. P., and McCormick, J. B. (1985) Descriptive analysis of Ebola virus proteins. *Virology*. **147**, 169–176
