# Supplementary material for: Impact of Ebola virus nucleoprotein on VP40 virus-like particle production: a computational approach
Source: Commun Biol. 2024 May 25;7:634. doi: 10.1038/s42003-024-06300-8 (PMC11128010; doi:10.1038/s42003-024-06300-8)
Supplement: Supplementary file 2 — Description of Additional Supplementary Files [file 42003_2024_6300_MOESM2_ESM.pdf]

Description of Additional Supplementary Files

File name: Supplementary Data 1-3
Description: Summary of calibration result for each round. Parameter ranges for each round. Chosen parameter sets for analysis and simulations.

File name: Supplementary Data 4-6
Description: Portion of cytoplasmic NP bound by VP40 in chosen parameter sets. Change of the average IB size in chosen parameter sets. Distribution of IB sizes in chosen parameter sets.

File name: Supplementary Data 7-8
Description: VLP production in chosen parameters. Parameter comparison between NP+VP40 and VP40 system.

File name: Supplementary Data 9-12
Description: VLP production in local sensitivity analysis for NP impact on filament dissociation constant. VLP production in local sensitivity analysis for NP impact on VLP budding rate constant. VLP production in local sensitivity analysis for NP/VP40 production ratio. NP and VP40 profiles in local sensitivity analysis for NP/VP40 production ratio.

File name: Supplementary Data 13
Description: VLP production when varying relative timing of expression of NP and VP40.

File name: Supplementary Data 14-15
Description: VLP production in fendiline treatment. VLP reduction at 24 and 48h in fendiline treatment.
